# Preferences for enhanced treatment options to address HIV care engagement among women living with HIV and perinatal depression in Malawi

**DOI:** 10.1186/s12889-023-16835-w

**Published:** 2023-10-04

**Authors:** Steve Mphonda, Josée Dussault, Angela Bengtson, Bradley N. Gaynes, Vivian Go, Mina C. Hosseinipour, Kazione Kulisewa, Anna Kutengule, Samantha Meltzer-Brody, Michael Udedi, Brian Pence

**Affiliations:** 1 UNC-Project Malawi, Private Bag A-104, Lilongwe, Malawi; 2 Department of Epidemiology, Gillings School of Global Public Health, University of North Carolina at Chapel Hill, Chapel Hill, NC USA; 3 Department of Health Behavior, Gillings School of Global Public Health, University of North Carolina at Chapel Hill, Chapel Hill, NC USA; 4 Department of Epidemiology, Brown School of Public Health, Brown University, Providence, RI USA; 5 Department of Psychiatry, UNC School of Medicine, University of North Carolina at Chapel Hill, Chapel Hill, NC USA; 6 Department of Medicine, UNC School of Medicine, University of North Carolina at Chapel Hill, Chapel Hill, NC USA; 7 Department of Psychiatry and Mental health, Kamuzu University of Health Sciences, Blantyre, Malawi; 8 Ministry of Health, Lilongwe, Malawi

**Keywords:** Perinatal depression, HIV, Mental health, Malawi

## Abstract

**Background:**

Option B + offers lifelong ART to pregnant or breastfeeding mothers, but postpartum loss to HIV care, partially driven by perinatal depression (PND), threatens the impact of this policy. This study aims to understand women’s and providers’ preferences for developing a feasible intervention to address PND and support engagement in HIV care among women living with PND and HIV.

**Methods:**

We conducted a total of 6 focus group discussions (FGDs) involving 4 clinics in Lilongwe District from December 2018 through February 2019. We conducted 2 FGDs each among 3 stakeholder groups: clinical staff, prenatal women, and postnatal women. Perinatal participants were living with HIV and screened positively for PND using the validated Edinburgh Postnatal Depression Scale (EPDS). Clinical staff were nurses who were trained antiretroviral therapy (ART) providers. Interviewers led FGDs in Chichewa using a semi-structured guide. Data were analyzed using deductive and inductive coding in NVivo 12 software.

**Results:**

Women favored ART linkage services, but providers said they already offered such services, with mixed results. Individual counselling was universally supported. A perceived benefit of group counselling was peer support, but there were concerns among women regarding confidentiality and stigma. Women liked mobile appointment reminders but identified low phone ownership as a barrier. Participants recommended home visits as an additional care engagement strategy. Women consistently discussed the need for social support from family members and friends to address PND and support engagement in HIV care.

**Conclusion:**

This study highlights the importance of peer encouragement to support perinatal HIV care engagement among women with HIV and PND. The results from this study can be used to support intervention development to increase HIV care engagement and improve long-term HIV outcomes in women with PND.

## Background

Antiretroviral therapy (ART) significantly reduces HIV viral load, disease progression and onward HIV transmission [1]. Today, throughout the sub-Saharan African region lifelong ART to all pregnant or breastfeeding mothers living with HIV is the standard of care for the prevention of mother-to-child transmission (PMTCT) and protection of long-term maternal health. In 2011, Malawi pioneered universal ART for pregnant and breastfeeding women with the Option B + program [2]. Option B + has the potential to reduce maternal mortality and mother-to-child HIV transmission in the region [3]. Life-long ART with sustained viral suppression also reduces the risk of HIV transmission to partners [4]. However, a lack of engagement in HIV care among women during the perinatal period threatens to limit the positive impact of Option B + on HIV care outcomes [5].

The lack in engagement in HIV care is partially driven by psychosocial issues, including perinatal depression (PND)(6). PND affects over 40% of women living with HIV (WLHIV) during the perinatal period(7). Evidence indicates that PND is more common among WLHIV as compared to those uninfected(8). Unemployment, unplanned pregnancy, poor ART adherence, and intimate partner violence (IPV) are understood to be associated with depressive symptoms among WLHIV(7). Additionally, specific psychosocial issues in pregnancy, including fear of infecting the newborn, stigma, insufficient social support, poverty, or disclosure concerns, further deepen women’s vulnerability to develop PND [9].

Retention in HIV care during the perinatal period is crucial for women, infants, and partners. Regular clinic visits are linked to improved ART adherence and viral suppression, which are essential to lower the risk of HIV drug resistance, HIV progression, and transmission to infants and partners [10]. Depression poses a challenge to retention in HIV care and ART adherence. Patients with depressive symptoms are less likely to adhere to medication than those without depressive symptoms [11]. The importance of treating depression and keeping women engaged in HIV care is therefore critical to reducing HIV transmission and improving HIV outcomes.

In sub-Saharan Africa, PND is substantially under-recognized and under-treated due to the unavailability of mental health services in primary health care settings and a critical shortage of mental health workers [12]. In Malawi, despite the high prevalence of PND and its detrimental consequences, perinatal women do not receive routine screening or treatment for depression during this high-risk period. We undertook this study with the aim of identifying promising strategies to address PND and improve HIV care engagement among perinatal WLHIV.

## Methods

### Context

In 2021, HIV prevalence in Malawi was at 8.9% among adults, with prevalence twice as high among women compared to men between ages 20 and 39 [13]. Between 2010 and 2021 new HIV infections have fallen by 61%, suggesting the country is on track to end the epidemic [14].

Mother to child transmission of HIV is still high with at least 2300 children acquiring HIV from their mothers in 2020 [15]. Malawi implemented Option B+, lifelong ART for pregnant and breastfeeding women, in 2011. Malawi achieved high coverage (98%) of routine antenatal HIV testing over 2011 and 2018 [16]. All women presenting to the ANC are offered routine HIV testing as an effort to limit mother-to-child transmission. All WLHIV qualify for lifelong ART, regardless of their CD4 count. ART is provided free of charge and integrated into antenatal care (ANC) in all public clinics in Malawi. Currently, screening for PND is not conducted routinely during antenatal or postpartum appointments.

### Study setting

Qualitative data were collected from 6 guided focus group discussions (FGDs) involving stakeholders from 4 clinics (2 urban, 2 rural) in Lilongwe District, Malawi from December 2018 to February 2019. We conducted 2 FGDs (1 at a rural site and 1 at an urban site) among each of the following groups: ART providers (nurses), prenatal women, and postnatal women.

### Study design and population

Women living with HIV were sensitized about the study and screened in antenatal and postnatal clinics using a validated Chichewa version of the Edinburgh Postnatal Depression Scale (EPDS)(17). All women 18 years and older with documented HIV infection and an EPDS score ≥ 10(18) were eligible for inclusion. Women at each clinic were screened consecutively until 6–8 women meeting inclusion criteria had consented to participate in the study. HIV status was confirmed using women’s health passports. Provider FGDs were conducted with a convenience sample of ART providers working in the antenatal and postnatal sections. We obtained written consent after assessing participant comprehension using a comprehension checklist. All study participants received transport reimbursement in the local currency equivalent to 10 US dollars.

### Data collection and analysis

We used FGDs to gather information on cultural beliefs on depression and HIV that affect attitudes and behavior. FGDs were led by a trained interviewer in Chichewa using a semi-structured guide that included questions on PND experiences, barriers to HIV care engagement, and two possible HIV care engagement strategies: postpartum ART linkage services and mobile phone appointment reminders. The FGDs also explored women’s preferences about receiving counselling for their depression, including individualized or group psychosocial counselling Participants were further asked to identify the cadre of healthcare worker who should provide counselling (nurse, doctor, or layperson). For ART linkage services, participants were asked if they thought it beneficial to have someone walk with a woman from the antenatal clinic to the ART clinic and introduce her to the process of how she will receive her ART postpartum, since pregnant women typically transition from receiving ART in antenatal clinics to ART clinics postpartum. Mobile reminders were described as text messages or phone calls to remind women to attend their ART clinic visits and collect their medication. FGD also offered women the opportunity to suggest their own ideas for engagement in care support. The FGD guide was developed by the research team and approved by the ethics committee. Each FGD had 6–8 participants and lasted approximately 90 min. We collected socio-demographic data for each participant before the commencement of the FGDs.

All FGDs were conducted in Chichewa and audiotaped. FGDs were then simultaneously transcribed and translated to English by AK. Transcribed FGDs were uploaded to NVivo 12 software [19] for data analysis, where they were then analyzed primarily by JD;BP;AB. Analysis and interpretation began during data collection as FGDs were transcribed and translated. Although weused a hybrid deductive (concept-driven) and inductive (data-driven) approach to coding the data [20], the coding approach relied heavily on the research questions and structure of the interview guides. Given that the FGDs were guided, with an interest to discover patient and provider preferences around specific interventions, the codebook was initially generated by JD;BP;AB based on the structure of the patient and provider guides. The codebook was then refined after reviewing the first few transcripts, ultimately incorporating additional subcodes that presented organically in the discussions. The codebook categorized data using descriptive codes [20]. JD;BP;AB;SM used thematic analysis methods [21] and created matrices to identify and analyze similarities and differences in each of the intervention-related themes. To best describe the qualitative analysis findings, illustrative examples are provided via direct quotes from transcripts.

### Ethics statement

This study was approved by the Institutional Review Boards at the University of North Carolina at Chapel Hill and at the Malawi National Health Sciences Research Committee.

## Results

### Demographic characteristics

Twelve HIV care providers and 25 peripartum women participated in the FGDs (Tables 1 and 2). The providers were all female with an average age of 36 and had attained 3 years or more of nursing training. All were trained in ART provision. Of the perinatal women, 13 were postnatal and 12 were antenatal. Perinatal women had an average age of 30 years. Most of these women were married with multiple children and had been living with HIV for more than two years.

**Table 1:** Patient characteristics (N = 25)

Women’s demographic information	N (%)
Mean age	30
Age range	19–46
Education	
*None*	3 (12%)
*≤ 8years*	16 (64%)
*Secondary*	6 (24%)
Marital status	
*Married*	21 (84%)
*Single*	1 (4%)
*Separated*	3 (12%)
Employment	
*Farmer*	8 (32%)
*Unemployed*	11 (44%)
*Business owner*	6 (24%)
Duration of HIV diagnosis	
*Diagnosed last 6 months ago* *6 months to 1 year*	5 (20%) 6 (24%)
*More than 1 year*	12 (48%)
Number of pregnancies	
*First*	4 (16%)
*Second*	4 (16%)
*Third*	6 (24%)
*Forth*	4 (16%)
*Fifth or more*	7 (28%)

**Table 2:** provider characteristics (N = 12)

*Position*	Urban N (%)	Rural N (%)
Nurse	6 (50%)	6 (50%)
Sex		
*Female*	6 (50%)	6 (50%)
Mean age	38	35
Age range	27–47	27–49
Training years		
*Less than 3 years*	0 (0%)	0 (0%)
*3 years and more*	6 (50%)	6 (50%)

### Preferred interventions to address PND

#### Individual counselling

All participants perceived counselling as an acceptable and feasible intervention for PND. However, some expressed fears about confidentiality in group counselling when sharing their problems, which could be a barrier to counselling. As one antenatal woman said:

*In a group, you cannot be free to express everything because you are ashamed; you don’t want other people to know some of the issues. So in a group, when you are presenting your problem, everyone knows your problem and knows that you are not able to handle your problem, while in one on-one-counseling, you are able to tell the counselor every detail of your problem.*

Individual counselling was favored more than group counselling, as participants feared that their private information could be discussed outside the group.

Most women, both antenatal and postnatal, agreed that they would be comfortable with individual counselling provided at the facility by a healthcare professional. Individual counselling was preferred because it is one-on-one and the client gets the counselor’s full attention, resulting in focused and individualized treatment. Another benefit that participants described was that the client would get direct feedback from the counselor regarding their progress.

Most of the healthcare providers also preferred individual counselling to group counselling. Similar to patients, the reasons providers gave for individual counselling were privacy and freedom for patients to express themselves. However, providers highlighted work overload and lack of space to conduct the counselling sessions as barriers to individual counselling. An HIV provider shared:

*The individual one is good because the person will verbalize all their concerns but maybe on the issue of staffing and space, it would mean that we will delay other activities because it can happen that there is just that one room and everyone is waiting for that one and the same staff are also responsible for other duties.*

Nurses were the first cadre suggested when women were asked, “Who would be the best to implement counselling.“ When asked if a layperson would be able to provide counselling, most providers were supportive, as long as the laypeople were well trained and supervised.

#### Group counselling

Group counselling was also highly rated among both women and HCWs.It allows for connection with other individuals who share similar struggles. This connection could foster a sense of belonging, identity, and relieve stress. Group counselling could offer a broader perspective on the issues being addressed in sessions as members can share their unique experiences with these issues. The group also offers a broader system of support for participants. A postnatal woman in the FGD offered:

*I feel like a group is better because you share experiences; it can happen that one has presented a problem and another person can share how she dealt with that similar problem. So you can see that among the people who are being counseled, they are encouraging each other. So, group counselling is helpful because the depression in the person can more easily be addressed than an individual counselling session.*

Providers saw group counselling as a means to address the challenges of low human resources in the facilities. They also saw the benefit in patients supporting one another. Several described an ideal situation in which women could receive both individualized counselling and group counselling. A provider in the FGD shared:

*I think what starts is the individual counselling and as you go along, when you see that the problems are persisting, the group counselling can work because she also hears her friends’ experiences and that can help her. At times, people feel they are all alone like they are the only ones experiencing that thing but when they are in a group; they are able to say my problems are better than hers. In that way, the person gets assisted.*

Group counselling was thought to be an effective way of addressing patient needs with limited human resource.

Concerns about group counselling involved stigma and lack of confidentiality. Some women and providers felt that patients would not be able to express themselves as fully, because they might feel shy and/or fear being stigmatized for what they share. An antenatal woman in the FGD shared:

*It cannot be very helpful because she is already isolating herself because she does not want people to know what is happening to her. She may not feel comfortable to be in a group because she cannot trust anyone in the group and as a result, she may not be able to express herself or she may not even present herself to the group because she does not want people to see her.*

Group counselling may also present challenges as the participants will have different values and cultures and may not always agree on certain aspects.

### HIV Engagement Strategy 1: Mobile reminders

FGD participants had varied views on the helpfulness of text message appointment reminders in increasing HIV care engagement in this population. Most believed that the mobile phone reminders could aid in the establishment of a routine for remembering to take their medication or report to the clinic for drug supply. As one antenatal woman shared:

*This can be helpful because many of us, when we are told the date, we can forget and even come days later: if they tell you the 8th you find that you come on the 15th to get the medication. When they ask you at the clinic you say that you had forgotten or you never knew the date, but if they were to remind you the date, you would come to get the medication.*

Despite the potential benefits of the reminders, participants identified lack of universal phone ownership as a barrier to such an intervention. Most of the women, especially in the rural areas, did not own a phone. Some have shared ownership with a family member or use their husband’s phone. For some, their households do not own a phone at all. Furthermore, they feared that there could be HIV-related stigmatization from an unintended disclosure of their HIV status via text message reminders.

Providers expressed concerns that receiving a text message would bring some misunderstanding in the family if their partners were not informed of the initiative. Participants discussed that lack of male partner involvement represents one of the main reasons for treatment refusal, delayed enrolment, dropout and low retention of pregnant and breastfeeding women. They mentioned that male involvement could be an important remedy to this problem. An HIV provider told the group,

*Like they have said that male involvement is really important, because the way you have put it that the messages would be like, ‘remember your health’. Some of the difficult men will still not understand that and what that means. They would just think that this is a way of hiding and think that that person has called her.*

### HIV Engagement Strategy 2: Postpartum ART linkage services

Most of the patient participants supported the idea of having someone help to coordinate the transition from receiving HIV treatment in an antenatal clinic to receiving HIV treatment in an ART clinic after the baby is born. It was mentioned that women have difficulty transitioning to the ART clinic because it is difficult to locate the clinic and most of the times the ART clinics are full and for a woman with a small baby it will be difficult to wait and access care. However, many providers discussed how linking the women to the ART clinic is already a current policy, yet it does not seem to address all women’s needs in making the transition. When FGD participants were asked which strategy was least important to adopt, providers often mentioned this strategy.

### Additional participant-suggested strategies

#### Social support

Throughout their discussions, participants consistently mentioned the benefits of the support one receives from their social network to manage their HIV and PND. Participants discussed that some people still think HIV diagnosis is a death sentence because there is no cure. Other people in the community are thought to be a source of discouragement, which makes it difficult for women to remain in HIV care and take medication. It was mentioned that the community looks at a person with HIV as promiscuous and unfaithful. Mothers also have fears about who is going to take care of their children if they die. Besides coping with stigma from friends and family, the women also experience their own concerns about transmitting the virus to the baby. The types of support mentioned included; emotional support (e.g., empathy, trust, or care), informational support (e.g., advice or information), and positive social interaction or social companionship (e.g., spending time with others). Sources of social support mentioned included husbands, other family members, friends, neighbors, and colleagues. As one postnatal woman said:

*There is need to have someone, whether a relative, a friend or even your husband to whom you can disclose that “I went to the antenatal clinic and I have been found HIV positive and I have been told that I should start taking ARVs. I am supposed to be taking the ARVs for the rest of my life and this means that I have to be going to the clinic to collect them.” When you open up like that, people are able to know your problem and assist you and you cannot be depressed.*

However, the women mentioned that fear of rejection and stigmatization make them choose not to reveal their HIV diagnosis. They said some family members had reacted badly after they had disclosed their HIV status. Sometimes people who are not part of one’s family may be the best to disclose to rather than a family member. One antenatal woman shared:

*It’s hard for us to be open and disclose to our families because they shout at us and you can’t even just tell them because they will start saying that everything that I use is stained with HIV. The neighbors who are not even your relatives are the ones that keep your secrets well…*.

### Home visits

The perinatal women organically introduced home visits as an important strategy for improving HIV care engagement in multiple FGDs. Participants suggested that women might not come to the clinic for various reasons like sickness, family issues, or transportation. Particularly in the final trimester of pregnancy, women identified walking far distances to attend a clinic as an important barrier to accessing care. Participants described how following up with perinatal women would enable the provider to determine the reasons a woman is not coming to the clinic. One postnatal woman shared:

*“The health workers should be able to visit the person who is sick and cannot maybe come to the clinic, this is in the case that this woman is pregnant. They can visit the person to see why the patient is not coming to the hospital”.*

Moreover, women suggested that if there were someone who could bring them their ART in third trimester and early postpartum or remind them to take medication and check if they have missed doses, they would be healthier and better informed. The women saw home visits as an important extension of social support.

## Discussion

In this qualitative study of perinatal women living with HIV and depression, and antenatal providers, there was universal support for developing interventions to better address PND among WLHIV. Counselling was identified as a feasible and acceptable intervention with barriers and challenges to individual vs. group counselling. The study also highlighted the limitations of mobile reminders as an HIV care engagement strategy and the potential benefits of social support and home visits to help women remain engaged in HIV care. These findings highlight the specific needs and preferences of women with HIV and PND, as well as their healthcare providers, to increase HIV care engagement and reduce PND. One key finding was that counselling provided by trained personnel was thought to be a feasible and acceptable intervention to help women struggling with PND and keep them engaged in HIV care. Both women and providers welcomed such an intervention to address the high burden of depression in this population. However, providers were wary of the challenges this extra task may bring due to inadequate human resource and lack of space within clinics. A study on such barriers in Zambia showed that lack of space and adequate staff caused a lapse in the quality of counselling provided [22]. For the successful implementation of a counselling intervention in this study population, dedicated space and personnel is needed to deliver confidential counselling.

Malawi, like many other sub-Saharan African countries, faces challenges in healthcare human resources generally and even greater challenges in mental healthcare personnel specifically [12] [23]. Task-shifting, which enables lay workers to provide services that would otherwise be restricted to professionals, can be a helpful way of reducing stress on a system that is already low in human resources. Task shifting is a logical strategy to address the scarcity of human resource for mental health [24]. Several interventions for improving mental health have proved to be effective using a task-shifting model. Lessons from LMICs highlighted the effectiveness of psychological treatments for common CMDs in adults delivered by trained and supervised community health workers or peers with no formal health-care role [25]. Psychosocial treatments were delivered by non-specialist providers through task sharing. A randomized controlled trial in Pakistan showed that a peer delivered psychosocial intervention, The Thinking Healthy Programme (THP) had a moderate effect on remission from perinatal depression over 6 months postpartum period [26]. The Friendship Bench is another intervention that utilizes lay health workers to implement brief, individual talking therapy sessions whose effectiveness has been demonstrated in several studies [27]. Friendship Bench therapy with education and support resulted in improved symptoms at 6 months among individuals of whom 86% were women [28] and group problem solving therapy provided by lay health workers was more effective than pharmacotherapy in the treatment of PND among women found to be depressed after childbirth [29].

Our findings provide insight into the feasibility and acceptability of mobile phone reminders to support engagement in HIV care among perinatal women. With the rapid increase of mobile phone subscriptions in low and middle-income countries, studies have used mobile phones to evaluate health outcomes and provide psychological support(30). Text messaging can change patients’ health behaviors, improve clinical outcomes, and increase healthcare utilization [31]. A meta-analysis found that HIV patients who received mobile phone reminders for their follow-up appointments were 2 times as likely to return to care as those who didn’t receive reminders(32). Compared to phone calls, text messages are more affordable with less interruption of recipients’ daily life. Use of mobile technologies may offer innovative and affordable approach to HIV prevention and care, particularly in resource-limited settings. However, in the present study, calls or text messages were thought to be helpful reminders to HIV care engagement among women, but participants were concerned about low phone ownership as a barrier to accessing mobile phone interventions. Moreover, participants perceived a risk of unintentional disclosure of their HIV status and stigma that may come from the frequent messages the woman is receiving.

Our results also revealed the need for social support to address PND among WLHIV. Women revealed that, after their HIV diagnosis, they have fears for themselves, their babies and their families. Emotional support, particularly acceptance and encouragement, may help to overcome these fears and help them start treatment and remain in HIV care. A study in Uganda showed that when individuals disclose their status, they seem to receive positive and supportive responses from family and friends to whom they disclose [33]. In another study, social support was found to have a significant association with reduced depression [34]. In our study, women suggested friends, relatives and, most importantly, their partners as a source of social support.

The findings from this study also highlighted the concerns women have that their partners will accuse them of infidelity leading to abandonment and discrimination. Attending the ART clinic may raise questions from the partner, especially for those who have not disclosed their HIV status. Disclosure of their HIV status to partners and positive support from the partners is essential for uptake of HIV care services in this population [35]. Without partner support, it is more difficult for women to adhere to recommended HIV treatment to reduce transmission of HIV to their infants, protect their own health, and ensure the health of their partner [36]. A study in Ethiopia showed that the proportion of HIV status disclosure to partners is relatively low despite its benefits [37]. There are unique risks associated with disclosing one’s status to one’s partner. Women mentioned loss of economic support, blame, abandonment, physical and emotional abuse, and discrimination as potential risks of disclosure.

Home visits were identified as another important strategy to help women remain engaged in HIV care. Both providers and women indicated that visiting patients in their homes would help women remain in care. Home visit objectives can include delivery of ART and psychosocial support. A study in Uganda showed that a home visit strategy resulted in significant increases in retention in care among pregnant and postpartum WLHIV and HIV-exposed infants [38]. Similarly in another study, home visits were associated with greater medication adherence in people living with HIV [39]. However, home visits need to be conducted in a manner that will not breach the patient’s confidentiality. Visiting the homes of women living with HIV may be challenging due to the stigma associated with HIV. In another study, community health workers made up stories of who they were and why they were visiting the patients to avoid status disclosure and stigma associated with HIV(40). If utilizing such a method, it is important that the health worker ask the patients how they would like the health worker to identify themselves when they visit the patients’ homes.

### Limitations

There are some limitations to this study. There could be social acceptability bias, because all women received counselling before the interview. We conducted our study in only one region of Malawi, in public health sector maternal and child health clinics, and, as such, some findings may not be applicable to other settings.

## Conclusions

This study highlights the importance of social support for perinatal HIV care engagement among women with HIV and PND. The study also highlighted peer support as a benefit of group counselling, but there were concerns regarding confidentiality and stigma that may prevent patients from expressing themselves fully. Mobile phone reminders were thought to be helpful in establishing a routine to take their medication and report to the clinic, but providers expressed concern that this may bring misunderstandings in the family if the partner is not aware of the initiative. Participants recommended home visits to provide medication and check if patients are missing doses as an additional care engagement strategy that would be an important extension of social support.The results from this study can be used to adapt interventions in order to increase HIV care engagement and improve long-term HIV outcomes in women with PND. Such efforts should strongly consider including depression counselling and strengthening social support. Mobile phone messaging may face challenges in similar populations related to phone ownership. Considering adaptations such as those presented here will be critical for addressing the mental health and HIV care engagement needs of perinatal women with depression and HIV in low-resource settings.

However, this study does not expand understanding of the needs for PND specifically or other social characteristics that may nuance engagement with different types of approaches to increase engagement in HIV care. We recommend further analysis of the relationship between PND and engagement and between wider cultural influences on PND as it may impact engagement.

## Data Availability

Data cannot be shared publicly because they contain sensitive patient information, as all prenatal and postnatal women are living with HIV and suffering from perinatal depression but are available from the corresponding author on reasonable request.

## Code Availability

NA.
